# Supplementary material for: Porcine sapovirus-induced RIPK1-dependent necroptosis is proviral in LLC-PK cells
Source: PLoS One. 2023 Feb 3;18(2):e0279843. doi: 10.1371/journal.pone.0279843 (PMC9897573; doi:10.1371/journal.pone.0279843)
Supplement: S1 Raw images — (PDF) [file pone.0279843.s002.pdf]

Western Blot Original Images

Fig 1B

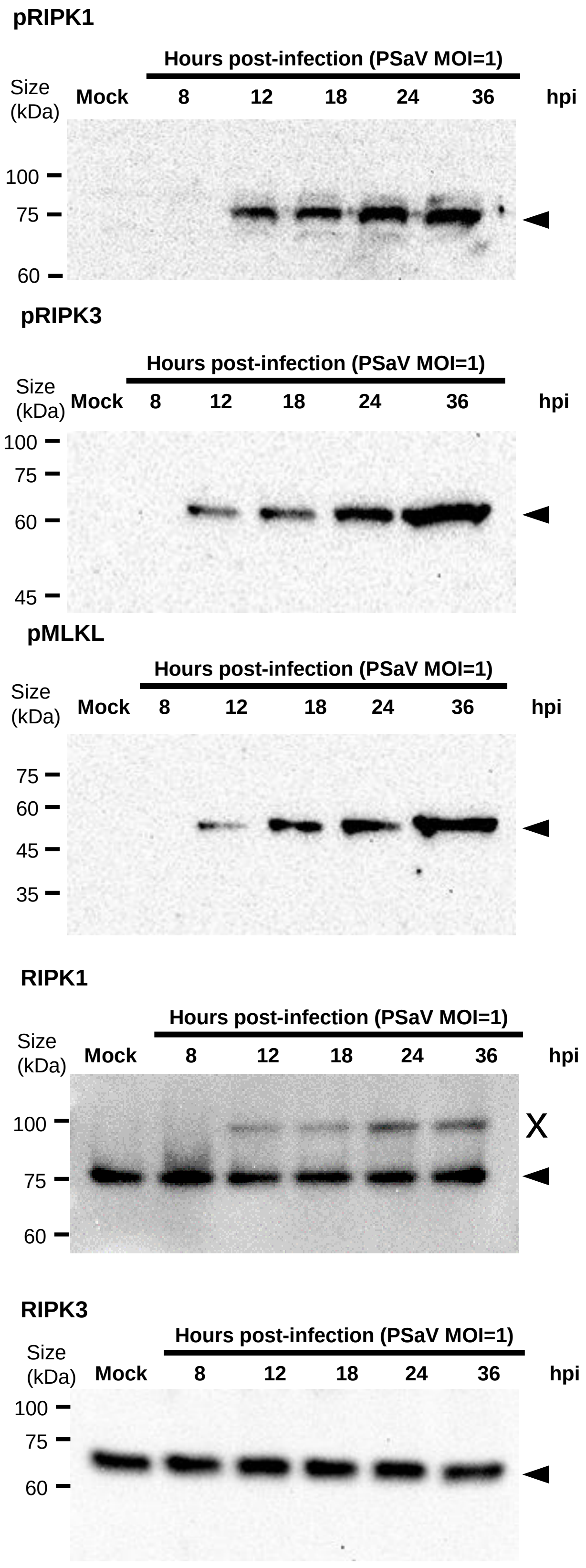
pRIPK1
Hours post-infection (PSaV MOI=1)
Size (kDa)
Mock
8
12
18
24
36
hpi
100
75
60
pRIPK3
Hours post-infection (PSaV MOI=1)
Size (kDa)
Mock
8
12
18
24
36
hpi
100
75
60
45
pMLKL
Hours post-infection (PSaV MOI=1)
Size (kDa)
Mock
8
12
18
24
36
hpi
75
60
45
35
RIPK1
Hours post-infection (PSaV MOI=1)
Size (kDa)
Mock
8
12
18
24
36
hpi
100
75
60
X
RIPK3
Hours post-infection (PSaV MOI=1)
Size (kDa)
Mock
8
12
18
24
36
hpi
100
75
60

Fig 1B

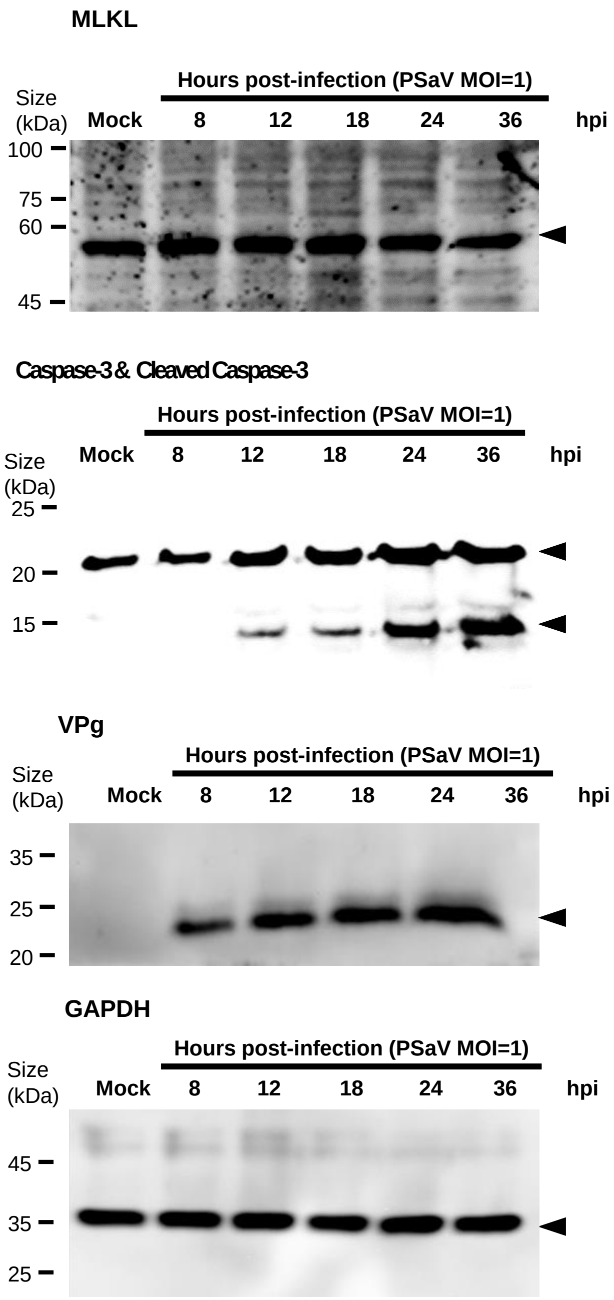
MLKL
Hours post-infection (PSaV MOI=1)
Size (kDa)
Mock 8 12 18 24 36 hpi
100
75
60
45
Caspase-3 & Cleaved Caspase-3
Hours post-infection (PSaV MOI=1)
Size (kDa)
Mock 8 12 18 24 36 hpi
25
20
15
VPg
Hours post-infection (PSaV MOI=1)
Size (kDa)
Mock 8 12 18 24 36 hpi
35
25
20
GAPDH
Hours post-infection (PSaV MOI=1)
Size (kDa)
Mock 8 12 18 24 36 hpi
45
35
25

FIG 4A

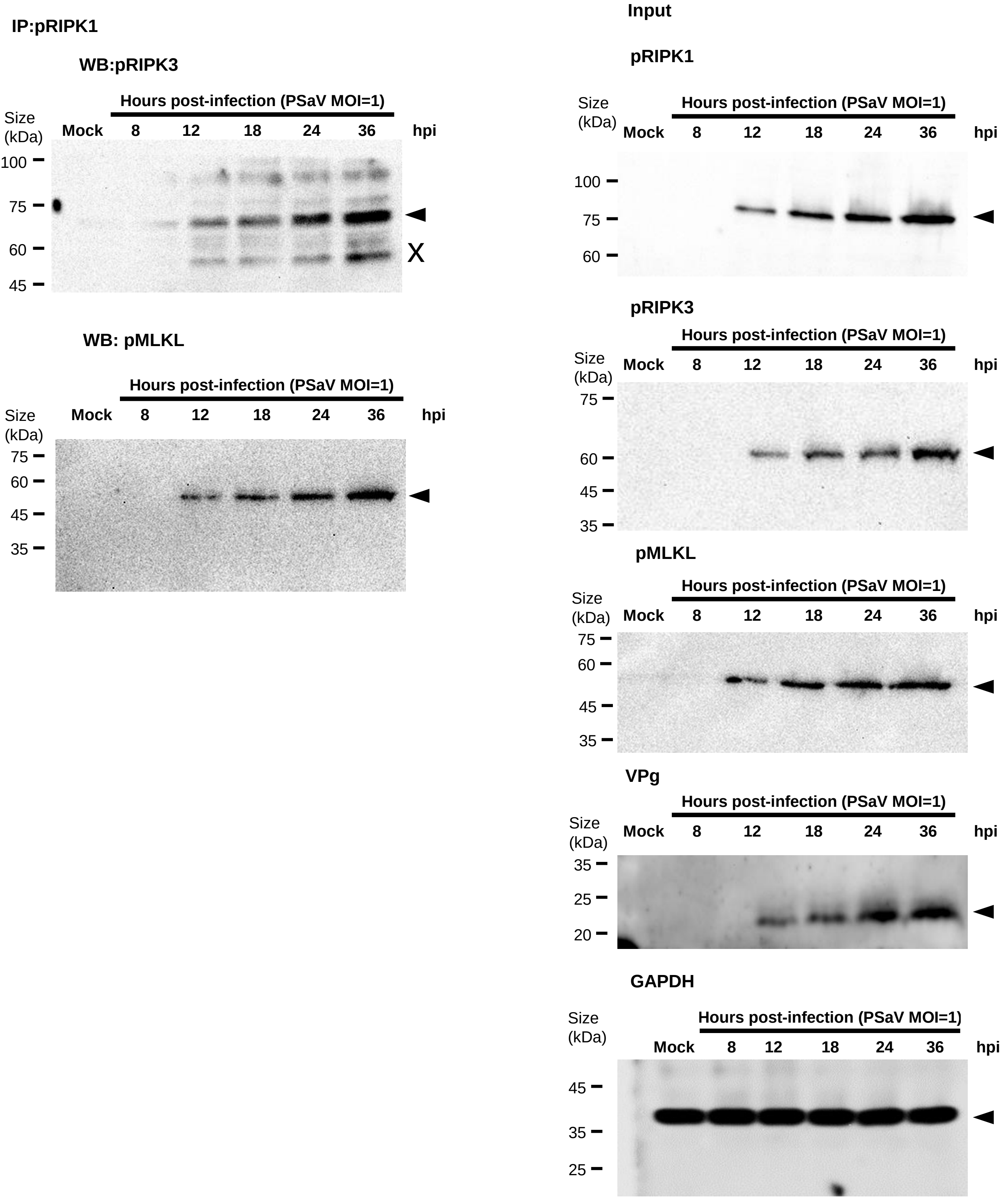
IP:pRIPK1
WB:pRIPK3
Hours post-infection (PSaV MOI=1)
Size (kDa)
Mock 8 12 18 24 36 hpi
100
75
60
45
X
WB: pMLKL
Hours post-infection (PSaV MOI=1)
Size (kDa)
Mock 8 12 18 24 36 hpi
75
60
45
35
Input
pRIPK1
Hours post-infection (PSaV MOI=1)
Size (kDa)
Mock 8 12 18 24 36 hpi
100
75
60
pRIPK3
Hours post-infection (PSaV MOI=1)
Size (kDa)
Mock 8 12 18 24 36 hpi
75
60
45
35
pMLKL
Hours post-infection (PSaV MOI=1)
Size (kDa)
Mock 8 12 18 24 36 hpi
75
60
45
35
VPg
Hours post-infection (PSaV MOI=1)
Size (kDa)
Mock 8 12 18 24 36 hpi
35
25
20
GAPDH
Hours post-infection (PSaV MOI=1)
Size (kDa)
Mock 8 12 18 24 36 hpi
45
35
25

FIG 6A

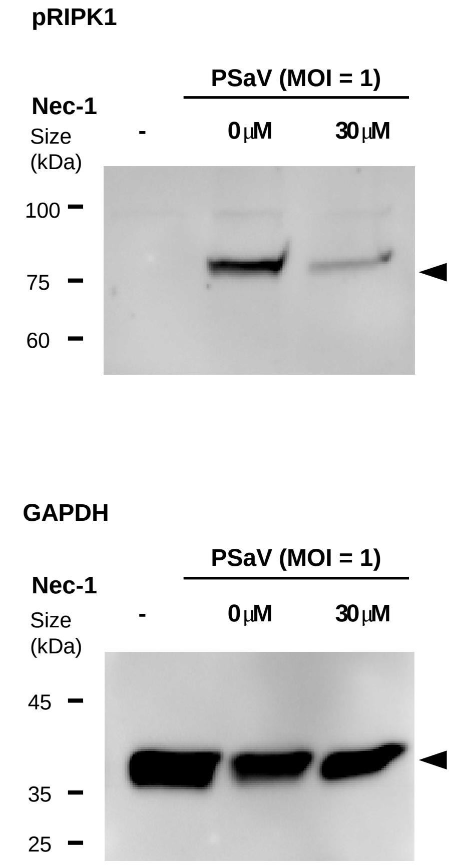

FIG 6B

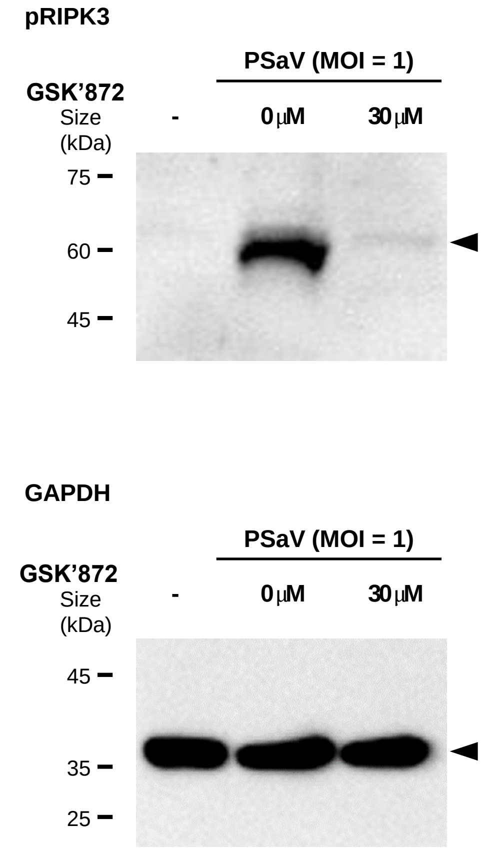

FIG 6C

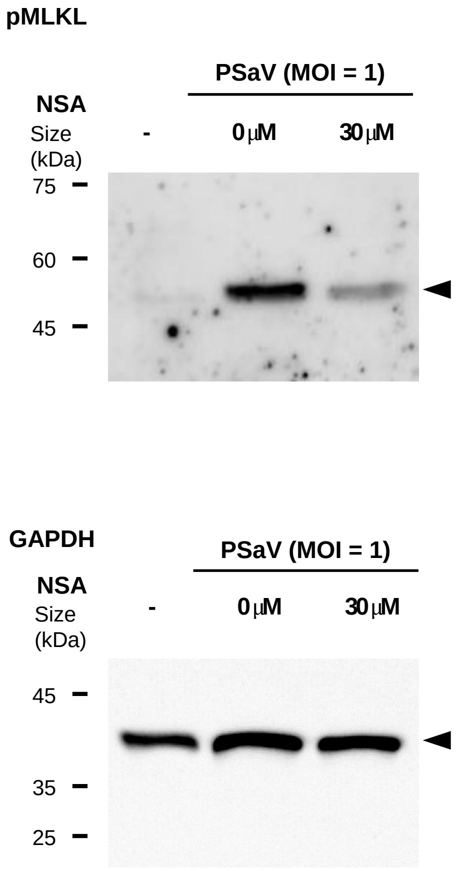

FIG 6D

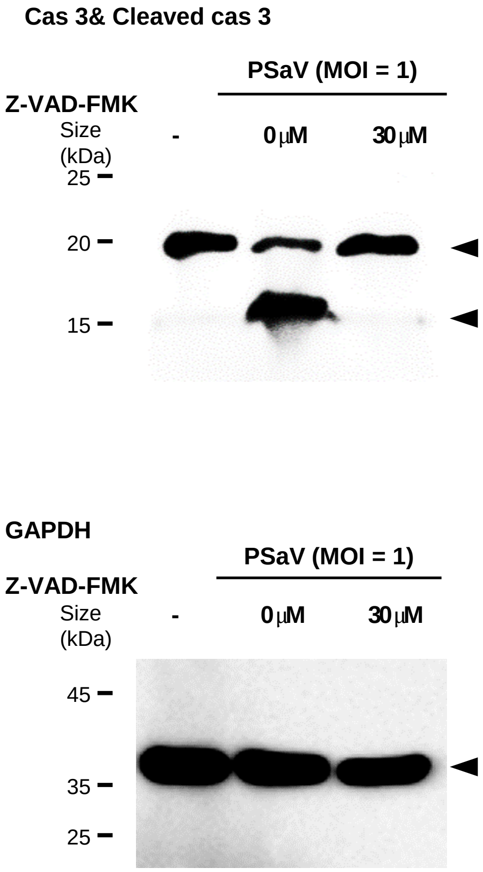

FIG 6D

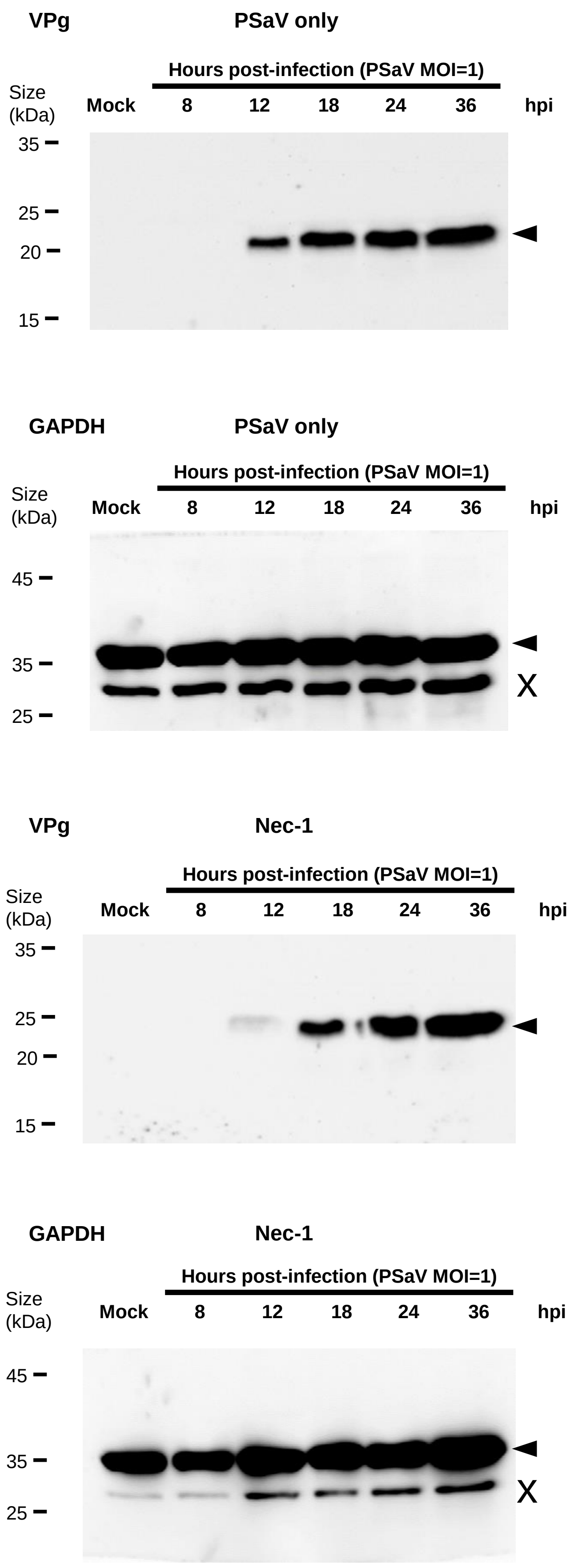

VPg
PSaV only
Hours post-infection (PSaV MOI=1)
Size (kDa)
Mock 8 12 18 24 36 hpi
35
25
20
15
GAPDH
PSaV only
Hours post-infection (PSaV MOI=1)
Size (kDa)
Mock 8 12 18 24 36 hpi
45
35
25
X
VPg
Nec-1
Hours post-infection (PSaV MOI=1)
Size (kDa)
Mock 8 12 18 24 36 hpi
35
25
20
15
GAPDH
Nec-1
Hours post-infection (PSaV MOI=1)
Size (kDa)
Mock 8 12 18 24 36 hpi
45
35
25
X

FIG 6E

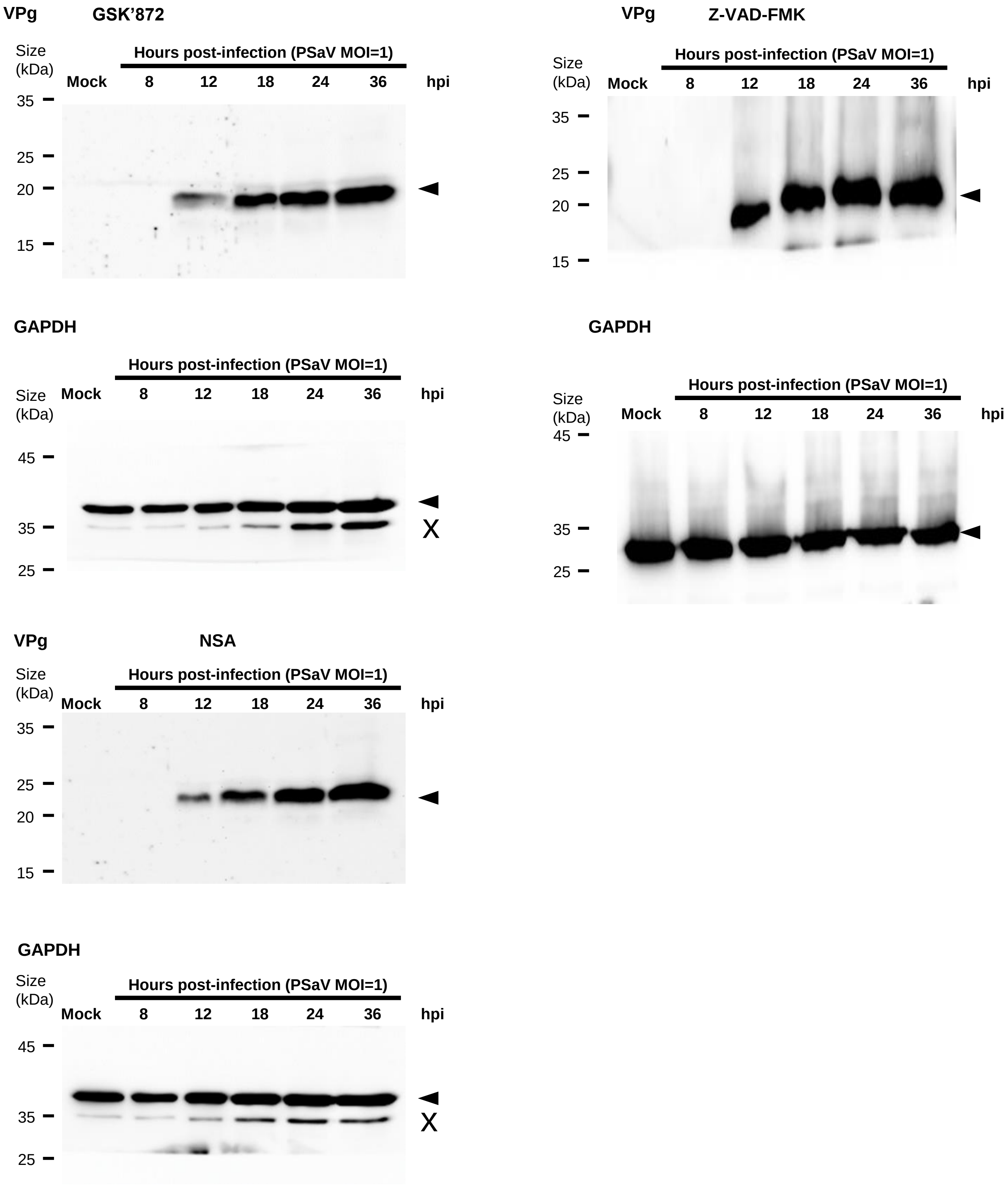
VPg
GSK'872
Size (kDa)
Hours post-infection (PSaV MOI=1)
Mock 8 12 18 24 36 hpi
35
25
20
15
VPg
Z-VAD-FMK
Size (kDa)
Hours post-infection (PSaV MOI=1)
Mock 8 12 18 24 36 hpi
35
25
20
15
GAPDH
Hours post-infection (PSaV MOI=1)
Size (kDa)
Mock 8 12 18 24 36 hpi
45
35
25
X
GAPDH
Hours post-infection (PSaV MOI=1)
Size (kDa)
Mock 8 12 18 24 36 hpi
45
35
25
VPg
NSA
Size (kDa)
Hours post-infection (PSaV MOI=1)
Mock 8 12 18 24 36 hpi
35
25
20
15
GAPDH
Size (kDa)
Hours post-infection (PSaV MOI=1)
Mock 8 12 18 24 36 hpi
45
35
25
X

FIG 7A

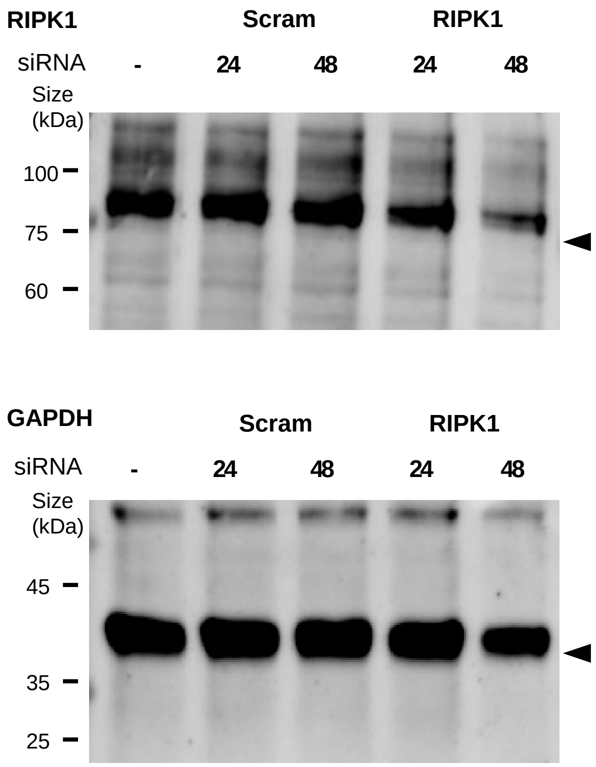

FIG 7C

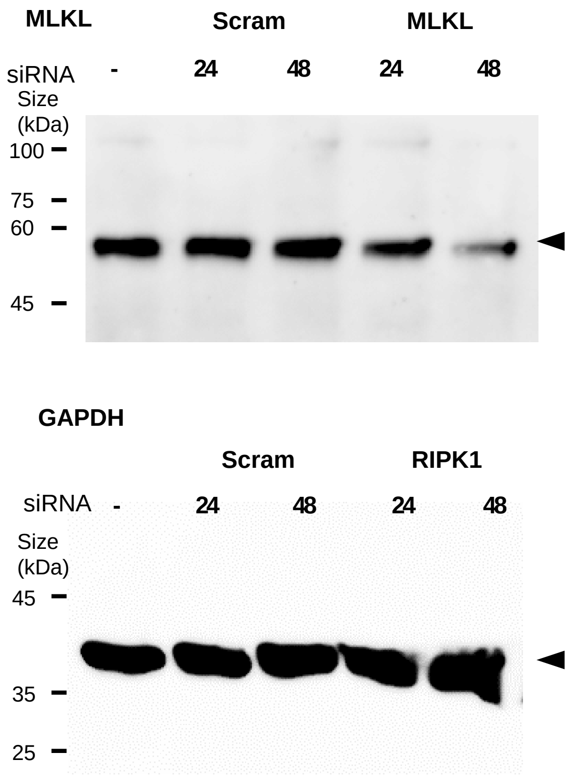

FIG 7B

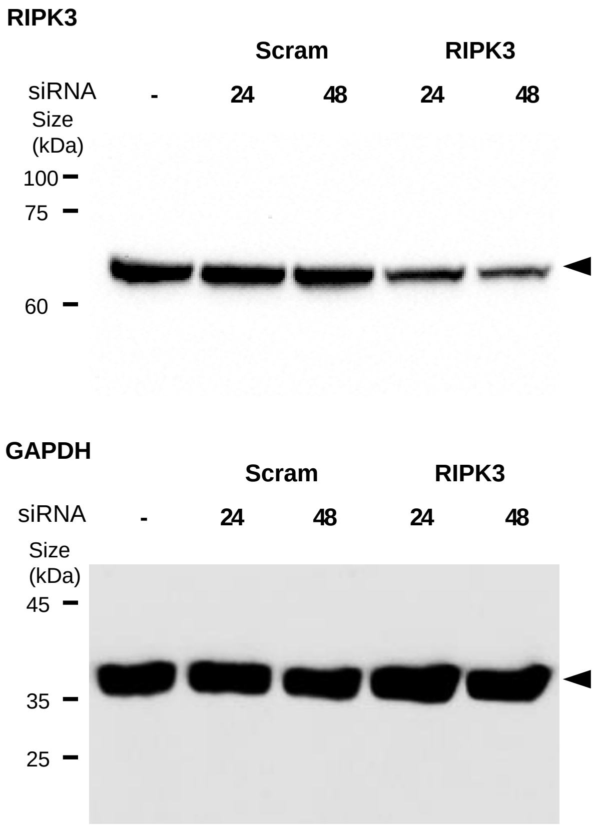

FIG 7D

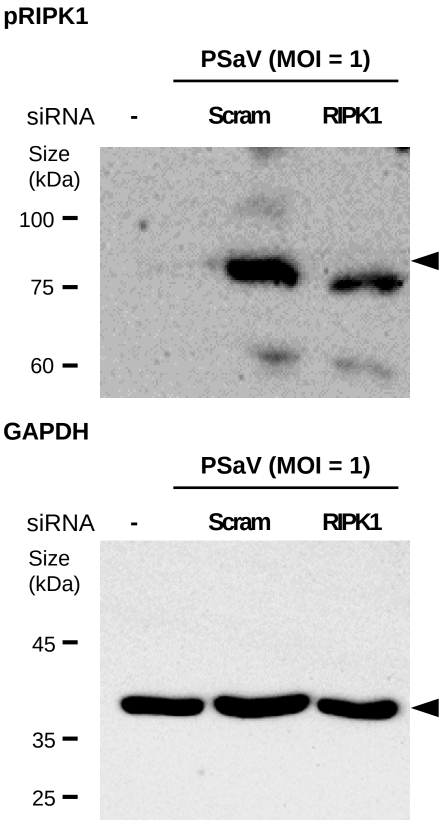

FIG 7F

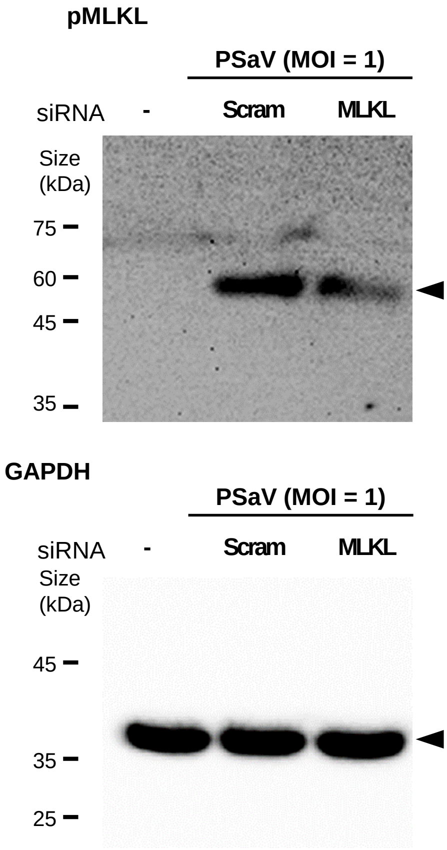

FIG 7E

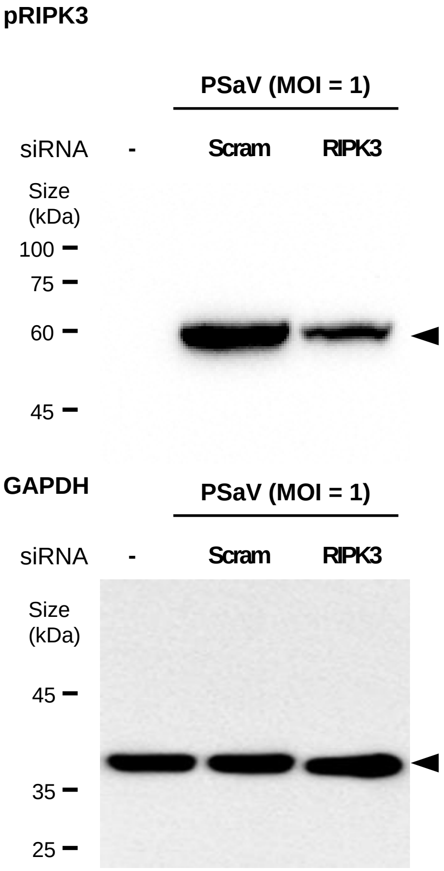

FIG 7G

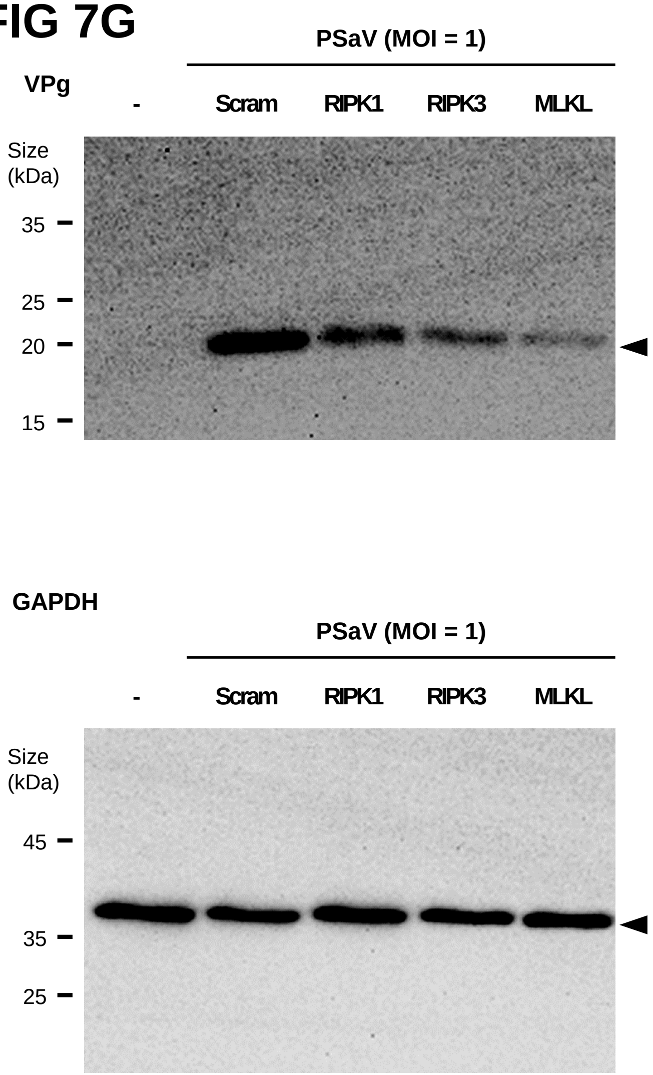
PSaV (MOI = 1)
VPg
-
Scram
RIPK1
RIPK3
MLKL
Size
(kDa)
35
25
20
15
GAPDH
PSaV (MOI = 1)
-
Scram
RIPK1
RIPK3
MLKL
Size
(kDa)
45
35
25
